# Evaluation of efficacy and safety of chemotherapy in the treatment of recurrent or resistant gestational trophoblastic neoplasia: A protocol for systematic review and meta-analysis

**DOI:** 10.1097/MD.0000000000027320

**Published:** 2021-10-08

**Authors:** Fang Luo, Li Li, Qing Gao, Yu-Xia Li

**Affiliations:** a Department of Gynecology, Wuhan Puren hospital, Wuhan, Hubei, China; b Department of pathology, wuhan puren hospital, Wuhan, Hubei, China; c Department of Gynaecology and Obstetrics, Wuhan Children's Hospita (Wuhan Maternal and Child Healthcare Hospital), Tongji Medical College, Huazhong University of Science & Technology, Wuhan, Hubei, China.

**Keywords:** chemotherapy, effectiveness, efficacy, gestational trophoblastic neoplasia, meta-analysis, recurrent, resistant, safety

## Abstract

**Background:**

Gestational Trophoblastic Neoplasia (GTN) is a spectrum of pregnancy-associated tumours emerging from placental tissue. Generally, GTN patients are considered to have a high rate of recovery. However, almost 25 per cent of GTN tumours resist, or have a high probability of relapsing following the first line of chemo treatment. Thus, tumours that resist or relapse requires salvage chemotherapy, sometimes accompanied by surgery. Globally, clinicians utilize a range of salvage regimens. Currently, ongoing debates are centred around choosing the best regimens in terms of safety and efficacy. Therefore, the current research aims to appraise the success and level of safeness using chemotherapy to treat patients with resistant or recurrent GTN.

**Methods:**

The authors will conduct a methodological exploration in online-based databases to find Randomized Controlled Trials related to the adoption of chemotherapy agents as treatment for resistant or recurrent GTN patients. The databases are as follows: EMBASE, PubMed, Cochrane Database Central, UpToDate, Chinese National Knowledge Infrastructure, Web of Science, and WanFang Database. The search will be limited to articles published in either English or Chinese. Moreover, the authors will also perform a search for ongoing trials on online-based clinical trial registries. Two independent authors will screen and select articles for review. A similar process will be followed by two independent authors to complete the extraction of data and evaluate the bias risk. In relevant cases, the authors will contract trial investigators to obtain related, unpublished data. The authors will use the random-effects model for pooling data in RevMan software (v5.3).

**Results:**

The present systematic review aims to evaluate the efficacy and level of safeness associated with using chemotherapy for resistant or recurrent GTN patients.

**Conclusion:**

The results of the proposed systematic analysis could summarize the most recent evidence for the use of chemotherapy agents on GTN patients.

**Ethics and dissemination:**

Since the proposed study uses pre-published data, an ethical approval is not required.

**Review registration number:**

Aug 25, 2021.osf.io/rgzbn. (https://osf.io/rgzbn/).

## 1 Introduction

Gestational trophoblastic disease alludes to a succession of interconnected tumours that emerge from placenta tissues. It includes premalignant disorders, benign molar pregnancies, choriocarcinoma, malignant disorders of invasive mole, epithelioid trophoblastic tumour, and placental site trophoblastic tumor.^[1–4]^ After pre-malignant disorders (whole and fractional hydatidiform mole), Gestational Trophoblastic Neoplasia (GTN) is prevalent in 15% to 20% and 1% to 4% of individuals, respectively, with local uterine invasion which can be accompanied by metastases.^[1,5]^ In most cases, GTN is an after effect of molar pregnancies. However, in some cases, it could occur after any gestation. In GTN, uterine haemorrhage is the most commonly observed symptom. However, extrauterine haemorrhagic cases could be the initial symptom in an individual with extrauterine spread: liver, lung, gastrointestinal tract, or brain.^[6–8]^ Recently, the wide accessibility of serum human chorionic gonadotropin measurement and first trimester ultrasound have resulted in changing the presentation of molar pregnancies. More specifically, it has changed from a condition associated in the second trimester to one in the first trimester. Therefore, most patients exhibit few symptoms at diagnosis.^[9]^ In general, molar pregnancies tend to resolve naturally after one or more uterine evacuations, which could be accompanied by chemotherapy. Invasive mole and Choriocarcinoma are the most prevalent types of GTN. Meanwhile, the occurrence of epithelioid trophoblastic and placental site trophoblastic tumours is rare.

In 2002, the International Society for the Study of Trophoblastic Diseases adopted the combination of the WHO-FIGO system.^[10,11]^ Accordingly, a score of six or less is representative of a low-risk. Meanwhile, a high-risk is classified by a score of seven or higher. Choriocarcinomas and invasive moles are tumours that are highly sensitive to chemo. As a result, the use of chemo agents can completely cure all lesions associated with a low level of risk and 80% to 90% of high-risk lesions. Still, nearly 25 per cent of cases will form resistance to the first line of treatment or will relapse. In such instances, salvage chemotherapy will be required. A second-line of chemotherapy encompassing paclitaxel/etoposide alternating with paclitaxel/cisplatin or bleomycin is extensively used for resistant or relapsing tumours.^[12,13]^ Reportedly, there is a 94 per cent overall cure rate for high-risk diseases.^[14]^ Over the last decades, there has been a substantial improvement in efficacious chemotherapy regimens, consequently improving the overall prognosis for GTN. In the majority of cases where females were treated with chemotherapy, medical professionals expect them to be long-term survivors. Therefore, to prove the prediction, the present systematic evaluation aims to evaluate the most effective chemotherapy agents to treat recurrent or resistant GTN patients and the efficacy and safety of these agents.

## 2 Objectives

To assess the efficacy and safety level of adopting different chemotherapy agents to heal recurrent or resistant GTN patients.

## 3 Methods

The present systematic review observes the Preferred Reporting Items for Systematic review and Meta-Analysis Protocol (PRISMA-P) statement. Moreover, the study has been registered under on OSF (https://osf.io/rgzbn/).^[15]^

### 3.1 Criteria for considering studies for this study

#### 3.1.1 Types of studies

Randomized Controlled Trials (RCTs). Only studies published in Chinese and English are considered for inclusion.

#### 3.1.2 Types of participants

Adult females (aged 18 and above) diagnosed with GTN will be considered. There aren’t any limitations in terms of ethnicity and age.

#### 3.1.3 Types of interventions

To compare treatment modalities: any chemo regimen against 1) radiotherapy, 2) different chemo regimen, 3) surgical treatment, and 4) chemoradiation. There aren’t any restrictions in terms of dose, duration, frequency, or combination.

#### 3.1.4 Types of outcome measures

The primary outcomes involve the complete survival rate, treatment failure, response rate, and remission/complete. Meanwhile, quality of life, progression-free survival, and adverse outcomes will be considered as secondary outcomes.^[16]^

### 3.2 Search methods for identifications of studies

#### 3.2.1 Electronic searches

The authors will systematically explore online-based databases to find RCTs related to adopting chemotherapy agents as treatment for resistant or recurrent GTN patients. The databases are as follows: EMBASE, PubMed, Cochrane Database Central, UpToDate, Chinese National Knowledge Infrastructure, Web of Science, and WanFang Database. The search will only consider articles written in either English or Chinese. The key search terms will be combined with terms associated with “chemotherapy,” “gestational trophoblastic neoplasia,” “randomized,” “randomized controlled trial.”

#### 3.2.2 Searching other resources

The authors will physically explore the references of suitable studies and related systematic reviews to seek out any other relevant articles.

### 3.3 Data collection and analysis

#### 3.3.1 Study selection

**Figure 1:**
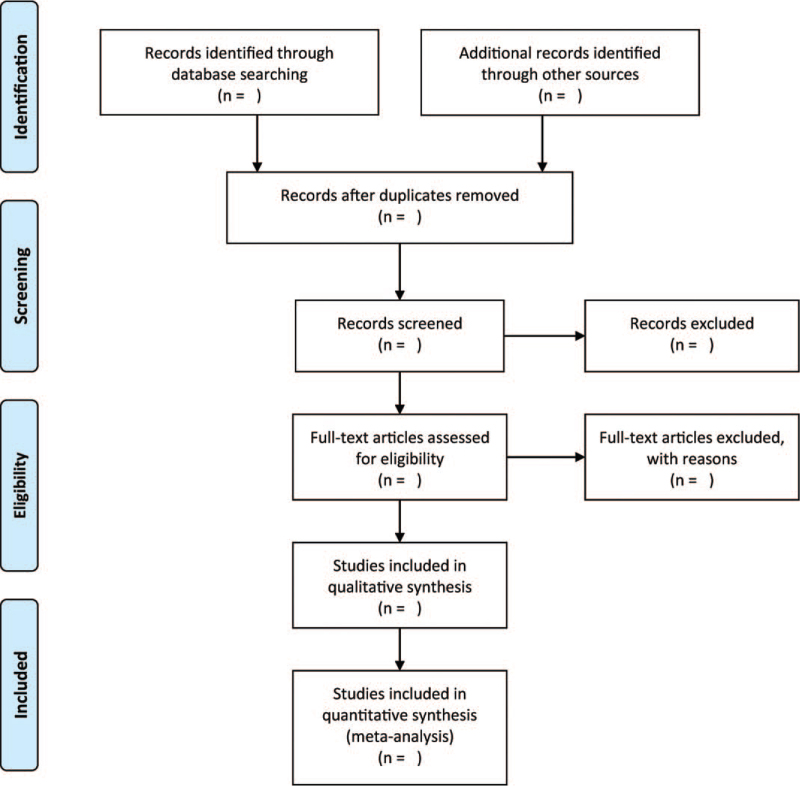
Flowchart of study selection process.

The screening and selection of articles for review shall be undertaken by a couple of independent authors. Besides, two independent authors will retrieve and assess the full-text based on the previously mentioned inclusion/exclusion criteria. Moreover, the same authors will examine the full-text of remaining articles to compile a final list of articles. Any disagreements during this process will be discussed with another independent author. Figure 1 shows the flow diagram related to the selection of study articles.

#### 3.3.2 Data extraction and management

The pertinent data of each research article that satisfy the inclusion criteria shall be compiled through a form to extract data. Two authors will autonomously extract all pertinent information. The extracted data will then be compared once the review concludes. A third independent author will be consulted to resolve all divergences. The extracted data will include name of authors, publication date, study design, intervention chemotherapy regimen, dose, duration, frequency, treatment duration, study population, age, histology, and outcome measures.

#### 3.3.3 Assessment of risk bias

The bias risk evaluation of the included RCTs will be based on the Cochrane Collaboration Tool.^[17]^ A third independent author will be consulted to resolve all divergences

#### 3.3.4 Measures of treatment effect

This systematic review shall adopt the effect treatment measures outlined:

In dichotomous results, the reviewers shall utilize the risk ratios and 95% confidence intervals;

For continuous results, the reviewers shall utilize the mean differences or standardized mean differences and 95% confidence interval;

For time-to-event data, the authors will employ the hazard ratio to facilitate comparison of the risk of fatality or progression of disease.

#### 3.3.5 Assessment of heterogeneity

Heterogeneity among the included articles shall be evaluated by visually inspecting the forest plots, estimating the heterogeneity percentage among trials that cannot be attributed to sampling variation, and through a official statistical test to assess the heterogeneity significance.^[18–20]^ In the case of any substantial heterogeneity, the authors will examine and report the plausible causes for the heterogeneity.

#### 3.3.6 Sensitivity analysis

The reviewers will perform a sensitivity analysis to appraise the robustness of the meta-analyses. It will be achieved by contrasting the results in all the trials before omitting trials of lesser systematic quality or those classified to pose an elevated bias risk.

## 4 Discussion

The planned analysis is the inaugural attempt to assess the efficacy and safeness of using different chemotherapy agents to treat resistant or recurrent GTN patients. The authors will endeavour to perform a comprehensive search of all related literature sources, including the grey sources to prevent missing any related study. A couple of autonomous authors will conduct all the procedures related to study selection, data extraction, and methodologic quality assessment. The authors will consult a third independent author to resolve all divergences. The results of the proposed systematic study will offer latest evidence on the efficiency and level of security associated with adopting different chemotherapy agents to cure resistant or recurrent GTN. Besides, the findings can also present helpful evidence for patients, clinicians, and direct future studies.

## Author contributions

Conceptualization: Fang Luo, Qing Gao, Yu-Xia Li.

Data curation: Fang Luo, Li Li, Qing Gao, Yu-Xia Li.

Formal analysis: Fang Luo, Li Li, Qing Gao.

Funding acquisition: Fang Luo, Li Li, Qing Gao, Yu-Xia Li.

Methodology: Fang Luo, Li Li, Qing Gao.

Project administration: Li Li, Qing Gao.

Resources: Fang Luo, Yu-Xia Li.

Software: Fang Luo, Li Li, Yu-Xia Li.

Supervision: Fang Luo, Qing Gao.

Validation: Fang Luo, Yu-Xia Li.

Visualization: Fang Luo, Li Li, Qing Gao, Yu-Xia Li.

Writing – original draft: Fang Luo.

Writing – review & editing: Yu-Xia Li.

## References

[1] Ngan HYS Seckl MJ Berkowitz RS. Update on the diagnosis and management of gestational trophoblastic disease. Int J Gynaecol Obstet 2018;143: Suppl 2: 79–85. 30306586 10.1002/ijgo.12615

[2] Lurain JR. Gestational trophoblastic disease II: classification and management of gestational trophoblastic neoplasia. Am J Obstet Gynecol 2011;204:11–8. 20739008 10.1016/j.ajog.2010.06.072

[3] Seckl MJ Sebire NJ Berkowitz RS. Gestational trophoblastic disease. Lancet 2010;376:717–29. 20673583 10.1016/S0140-6736(10)60280-2

[4] Abu-Rustum NR Yashar CM Bean S. Gestational trophoblastic neoplasia, version 2.2019, NCCN clinical practice guidelines in oncology. J Natl Compr Canc Netw 2019;17:1374–91. 31693991 10.6004/jnccn.2019.0053

[5] Berkowitz RS Goldstein DP. Clinical practice. Molar pregnancy. N Engl J Med 2009;360:1639–45. 19369669 10.1056/NEJMcp0900696

[6] Ahamed E Short D North B Savage PM Seckl MJ. Survival of women with gestational trophoblastic neoplasia and liver metastases: is it improving? J Reprod Med 2012;57:262–9. 22696824

[7] Savage P Kelpanides I Tuthill M Short D Seckl MJ. Brain metastases in gestational trophoblast neoplasia: an update on incidence, management and outcome. Gynecol Oncol 2015;137:73–6. 25598530 10.1016/j.ygyno.2015.01.530

[8] Frijstein MM Lok C van Trommel NE. Lung metastases in low-risk gestational trophoblastic neoplasia: a retrospective cohort study. Bjog 2020;127:389–95. 31794098 10.1111/1471-0528.16036

[9] Elias KM Berkowitz RS Horowitz NS. State-of-the-Art workup and initial management of newly diagnosed molar pregnancy and postmolar gestational trophoblastic neoplasia. J Natl Compr Canc Netw 2019;17:1396–401. 31693988 10.6004/jnccn.2019.7364

[10] Current FIGO staging for cancer of the vagina, fallopian tube, ovary, and gestational trophoblastic neoplasia. Int J Gynaecol Obstet 2009;105:03–4. 10.1016/j.ijgo.2008.12.015 19322933

[11] Ngan HY Bender H Benedet JL Jones H Montruccoli GC Pecorelli S. Gestational trophoblastic neoplasia, FIGO 2000 staging and classification. Int J Gynaecol Obstet 2003;83: Suppl 1: 175–7. 14763174 10.1016/s0020-7292(03)90120-2

[12] Bouchard-Fortier G Ghorani E Short D. Following chemotherapy for gestational trophoblastic neoplasia, do residual lung lesions increase the risk of relapse? Gynecol Oncol 2020;158:698–701. 32654764 10.1016/j.ygyno.2020.06.483

[13] Lurain JR Nejad B. Secondary chemotherapy for high-risk gestational trophoblastic neoplasia. Gynecol Oncol 2005;97:618–23. 15863169 10.1016/j.ygyno.2005.02.004

[14] Agarwal R Alifrangis C Everard J. Management and survival of patients with FIGO high-risk gestational trophoblastic neoplasia: the U.K. experience, 1995–2010. J Reprod Med 2014;59:07–12. 24597279

[15] Moher D Shamseer L Clarke M. Preferred reporting items for systematic review and meta-analysis protocols (PRISMA-P) 2015 statement. Syst Rev 2015;4:01. 10.1186/2046-4053-4-1 PMC4320440 25554246

[16] Cancer N. Common Terminology Criteria for Adverse Events (CTCAE) v4.0. 2009.

[17] Higgins JP Altman DG Gøtzsche PC. The Cochrane Collaboration's tool for assessing risk of bias in randomised trials. BMJ 2011;343:d5928. 22008217 10.1136/bmj.d5928 PMC3196245

[18] Higgins JP Thompson SG Deeks JJ Altman DG. Measuring inconsistency in meta-analyses. BMJ 2003;327:557–60. 12958120 10.1136/bmj.327.7414.557 PMC192859

[19] Mantel N Haenszel W. Statistical aspects of the analysis of data from retrospective studies of disease. J Natl Cancer Inst 1959;22:719–48. 13655060

[20] DerSimonian R Laird N. Meta-analysis in clinical trials revisited. Contemp Clin Trials 2015;45 (Pt A):139–45. 26343745 10.1016/j.cct.2015.09.002 PMC4639420

